# Eribulin shows high concentration and long retention in xenograft tumor tissues

**DOI:** 10.1007/s00280-017-3369-7

**Published:** 2017-06-29

**Authors:** Michiko Sugawara, Krista Condon, Earvin Liang, Christopher DesJardins, Edgar Schuck, Kazutomi Kusano, W. George Lai

**Affiliations:** 1 Medical Communication Section, Medical Division, Eisai Co., Ltd., Nishigoken-cho 13-1, Shinjuku-ku, Tokyo, 162-0812 Japan; 2 DMPK-Andover, Biopharmaceutical Assessments, Eisai Inc., Ltd., Massachusetts, USA; 3 Tsukuba Research Laboratories, Eisai Co., Ltd., Ibaraki, Japan

**Keywords:** Eribulin, Tumor, Pharmacokinetics, Xenograft

## Abstract

**Purpose:**

Eribulin, a synthetic analog of the natural product halichondrin B, is a microtubule dynamics inhibitor. In this study, we report the pharmacokinetic profiles of eribulin in mice, rats, and dogs following intravenous administrations with optimized and validated bio-analytical methods.

**Methods:**

Eribulin was administered at 0.5 and 2 mg/kg in mice, 0.5 and 1 mg/kg in rats, and 0.08 mg/kg in dogs. Tumor and brain penetration of eribulin was also evaluated in LOX human melanoma xenograft models. Concentrations in plasma, tumor, and brain were measured by the LC–MS/MS method.

**Results:**

The profiles of eribulin were characterized by extensive distribution, moderate clearance, and slow elimination in the three species. The pharmacokinetics are linear in mice and rats. In xenograft mice, the penetration into the brain was low, as expected, since eribulin is a P-glycoprotein substrate. In contrast to disposition in brain, the exposure of eribulin was approximately 20–30 times higher in tumor than that in plasma and half-lives were 17.8–35.9 h after both single and multiple dose regimens.

**Conclusions:**

Eribulin was distributed rapidly and eliminated slowly in mice, rats, and dogs. The exposure of eribulin was approximately 20–30 times higher in tumor than in plasma in xenograft mice. These results might be caused by eribulin’s mechanism of action including increased perfusion in tumor by vascular remodeling effect.

## Introduction

**Fig. 1:**
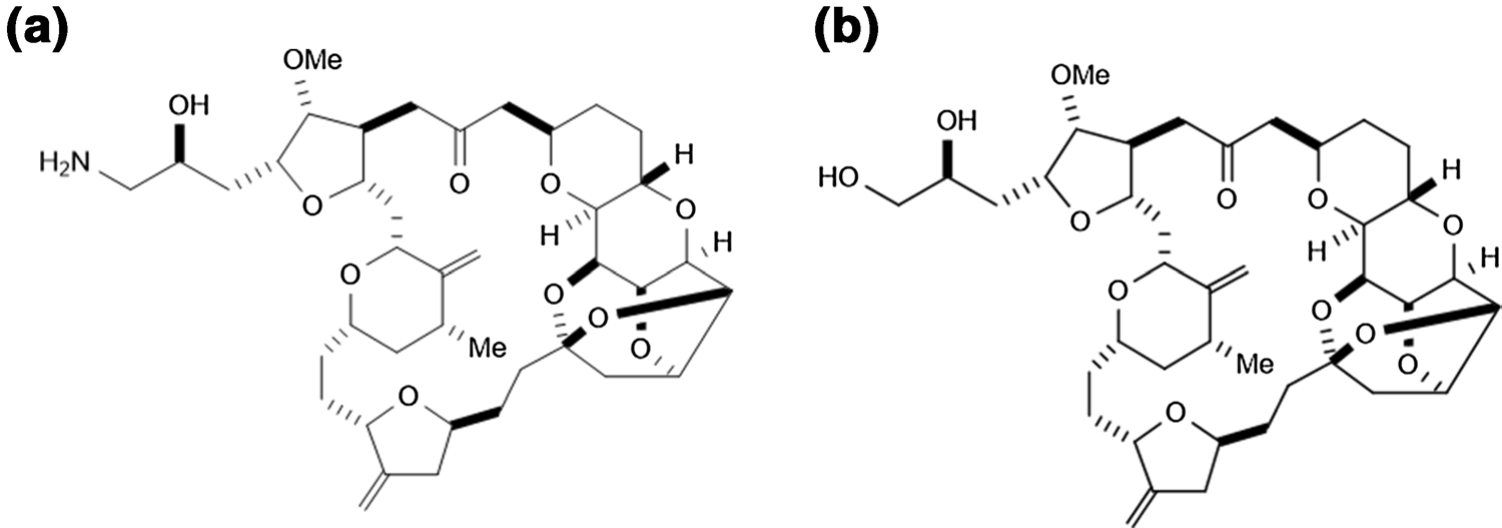
Chemical structures of eribulin and IS. **a** Eribulin. Molecular weight 729.90. **b** IS. Molecular weight 730.88

Eribulin (Fig. 1), a synthetic analog of the natural product halichondrin B, is currently being used as an anti-cancer agent. Eribulin has sub-nanomolar growth inhibitory activities in vitro against numerous human cancer cell lines as well as marked in vivo activities at 0.1–1 mg/kg against human xenografts via irreversible mitotic blockade [1, 2]. Eribulin’s mode of action is distinct from other tubulin inhibitors, and it involves binding to specific sites on the growing positive ends of microtubules to inhibit their growth [2–4]. Eribulin also induces vascular remodeling, suppresses migration and invasion of cancer cells, and reverses the epithelial-to-mesenchymal transition in cancer cell lines [5–7].

Eribulin has been approved for treatment of metastatic breast cancer who has previously received at least two chemotherapeutic regimens including an anthracyclines and a taxane and unresectable or metastatic liposarcoma who have received a prior anthracycline-containing regimen in the US. Phase III trials revealed improvement in overall survival in such patients treated with eribulin, compared with the conventional treatments [8–10]. Pharmacokinetic (PK) profiles of eribulin have been reported in clinical studies. The PK profile of eribulin was characterized by an extensive volume of distribution, slow-to-moderate clearance, and slow elimination. Area under the plasma concentration versus time curve (AUC) and maximum plasma concentration (*C*_max_) increased in almost direct proportion to the dose of eribulin (0.25–4 mg/m^2^) [11–13]. A mass balance study with radiolabeled eribulin in patients with advanced solid tumors showed that more than 80% of administered radioactivity was excreted in feces, where unchanged eribulin was 61.9%, whereas renal excretion of eribulin was 8.9% [14]. These results indicate that metabolism and renal clearance contribute to the elimination of eribulin in a minor way.

Preclinical studies have shown that higher brain penetration of eribulin was observed in ATP-binding cassette subfamily B (ABCB) member 1a-deficient mice, a subpopulation that naturally lacks the ABCB1a-encoded P-glycoprotein (P-gp), compared with the wild-type mice, suggesting that eribulin is likely to be a P-gp substrate [15].

The efficacy and toxicity of anti-cancer drugs in the treatment of tumors are highly dependent on the ability of the drugs to distribute into tumor tissue at the optimal therapeutic dose. In a preclinical study, eribulin exhibited wide in vivo therapeutic windows [1]. In phase II and III clinical studies, eribulin showed a significant and clinically meaningful improvement in overall survival of patients with metastatic breast cancer compared with treatment of physician’s choice [8], with manageable safety profiles. The most common grade 3/4 adverse events (AE) were neutropenia (45.2%), leukopenia (13.9%), and asthenia/fatigue (8.8%). Although peripheral neuropathy was a common AE leading to treatment discontinuation, the peripheral neuropathy typically improved in later cycles. Thus, eribulin showed balanced profile of efficacy and safety in preclinical and clinical settings.

There has not been a report of preclinical PK profiles of eribulin in tumor-bearing mice or in other preclinical species. In this study, PK profiles of eribulin in mice, rats, and dogs following intravenous administration were investigated with optimized and validated bio-analytical methods. In addition, brain and tumor penetration of eribulin were evaluated in a xenograft model to further characterize anti-tumor activity in terms of its disposition.

## Materials and methods

### Chemicals and reagents

Eribulin and the internal standard (IS, ER076349) were synthesized at Eisai Research Institute (ERI, Andover, MA) (Fig. 1). All reagents used in this study were of analytical grade. Mouse, rat, and beagle control plasma containing EDTA as the anticoagulant were purchased from BioreclamationIVT (Westbury, NY), Cocalico Biologicals Inc. (Reamstown, PA), or Biological Specialty Corp. (Colmar, PA).

### PK studies in mice, rats, and dogs

Male BALB/c mice (22–24 g) and jugular vein cannulated male Sprague–Dawley rats (226–250 g) were purchased from Charles River Laboratories (Wilmington, MA). Male purebred beagle dogs (approximately 8 kg and 6–8 months old) were selected from the stock colony in Covance (Princeton, NJ). Dosing solutions of eribulin were freshly prepared by dissolving eribulin in deionized water, and administered intravenously via tail veins in mice and rats and cephalic veins in dogs. The doses studied were 0.5 and 2 mg/kg in mice, 0.5 and 1 mg/kg in rats, and 0.08 mg/kg in dogs. According to the conversion of doses based on body surface area [16], the doses in this study would be equivalent to the 1.4 mg/m^2^ clinical dose of eribulin.

Blood specimens were collected from each species at predetermined time points (5, 15, 30 min, and 1, 2, 4, 8, 12, 24, and 48 h for mice; 5, 7.5, 10, 15, 25, 40 min, and 1, 2, 4, 6, 8, 12, 24, and 48 h for rats; 5, 7.5, 10, 15, 25, 40 min, and 1, 2, 4, 6, 8, 12, 24, 36, and 48 h for dogs) and stored in tubes containing sodium EDTA as anticoagulant. Plasma levels of eribulin from all species were determined by liquid chromatography–tandem mass spectrometry (LC–MS/MS).

All the studies using laboratory animals were approved either by the Eisai (Andover, MA) Institutional Animal Care and Use Committees (IACUCs) or the IACUC of the contract research organization that conducted the study, and adhered to all of the applicable institutional and governmental guidelines for the humane care and use of laboratory animals.

### Tumor and brain penetration in xenograft model

Tumor and brain penetration of eribulin was evaluated in LOX human melanoma xenograft models as previously described [1]. In brief, female Ncr LOX tumor-bearing nu/nu mice (*n* = 3/time point/dose group) were given a single 1.0 or 2.0 mg/kg intravenous dose of eribulin or 0.5 or 1.0 mg/kg intravenously once every 2 days for three doses (Q2Dx3). Blood, tumor, and brain tissues were collected from each animal at predetermined time points (15, 30 min, and 1, 2, 4, 8, 12, 24, and 36 h postdose). Concentrations of eribulin in brain, tumor, and plasma were determined by LC–MS/MS.

### Analysis of plasma samples

Analysis of eribulin in mouse, rat, and dog plasma was performed using a validated LC–MS/MS method with modifications for human plasma as previously described [17]. In brief, an aliquot of plasma sample was mixed with 500 ng/mL of IS and extracted with a C8 SPE cartridge. The eluent was collected and evaporated to dryness in a 35 °C water bath under nitrogen, and then reconstituted with methanol, water, and formic acid (50:50:1; by volume). Each sample was analyzed on a Quattro Ultima Micromass triple quadrupole mass spectrometer (Micromass Limited, Beverly, MA) using electrospray ionization under positive ion mode. Eribulin was monitored at precursor ion as a mass-to-charge ratio (*m/z*) at 730.4 and product ion *m/z* at 712.5, and IS was monitored at precursor ion *m/z* at 731.4 and product ion *m/z* at 681.5. Mobile phases A and B consisted of acetonitrile, water, and formic acid (13:87:0.1; by volume), and acetonitrile, tetrahydrofuran, and formic acid (70:30:0.1; by volume), respectively. Aliquots were injected onto a C18 MetaChem Polaris 3 μm, 30 mm × 2.0 mm column (Agilent, Santa Clara, CA). Retention times for eribulin and IS were 4.2 and 4.5 min, respectively. Calibration and quality control standards were freshly prepared in each analytical run using appropriate blank plasma. Peak areas of eribulin and IS were calculated and integrated using QuanLynx 3.5 (Micromass Limited; Beverly, MA).

### Analysis of brain and tumor samples

LC–MS/MS analyses of eribulin in brain and tumor samples were performed. In brief, tumor or brain tissue was prepared by homogenizing the specimen with three times its weight of phosphate-buffered saline using a Polytron PT 1200 tissue homogenizer (Kinematica AG, Lucern, Switzerland). The brain or tumor homogenate was vortexed with IS and 0.1 N sodium hydroxide in a polypropylene tube. Following addition of nano-pure water and further mixing, a solution of ethyl acetate, methanol, and ethanol (90:5:5, by volume) was added into the mixture, shaken horizontally, and then centrifuged at 4 °C. An aqueous phase of the mixture was frozen in a dry ice/isopropyl alcohol bath to retrieve the organic phase. The collected organic phase was transferred to a polypropylene tube and dried under nitrogen at 35 °C. The residue was then reconstituted with methanol/water (1:1, by volume) containing 0.1% formic acid. A Shimadzu Co-Sense high-performance liquid chromatography system (Shimadzu Scientific Instruments, Columbia, MD) was used for gradient elution of eribulin. Aliquots were injected onto a Polaris C18-A column (30 mm × 2.0 mm) with a constant temperature of 30 °C. Retention times for eribulin and IS were approximately 4.5 min and 4.6 min, respectively. Detection of the eluents was conducted using an API4000 triple quadrupole mass spectrometer (Sciex, Framingham, MA) using electrospray ionization under positive ion mode. Eribulin was monitored at precursor ion *m/z* 730.5 and product ion *m/z* 712.5, and the IS was monitored at precursor ion *m/z* 731.5 and product ion *m/z* 681.5.

### PK analysis

PK parameters of eribulin were estimated through noncompartmental analysis using WinNonLin 4.0.1 (Certara, Princeton, NJ). Eribulin plasma concentrations below the quantification limit were left blank at time 0 and treated as 0 at other time points. Nominal time was used for all analyses with the exception of actual time deviations greater than 10% for sampling time prior to 24 h, or 1 h for sampling time post 24 h. The actual time was used for those cases. For mice PK, the parameters were calculated based on mean concentration of each time point, because the blood at each point was collected from independent animals. Plasma concentrations and PK parameters of eribulin are expressed as mean or mean ± standard deviation (SD) using Excel 2000 (Microsoft, Redmond, WA) in rats and dogs. Penetration of eribulin into tumor and brain was assessed by the tumor penetration index (TPI) and brain penetration index (BPI), respectively, which were calculated as the ratio of the AUC in the tumor or brain to that in the plasma.

### Statistical analysis

Statistical analysis was performed using SigmaStat 2.03 (SPSS Inc.; Chicago, IL). One-way analysis of variance was used to examine the concentration effects on PK parameters among groups with *P* value less than 0.05 considered statistically significant. Outliers when applicable were rejected based on the Dixon’s criteria [18].

## Results

### Linearity and lower limit of quantification

Plasma levels of eribulin were linear from 0.5 to 500 ng/mL for all species studied. Regression coefficients of the calibration curves were greater than 0.979 in all matrices investigated. The quantification limit was 0.5 ng/mL for plasma and 2 ng/g for tumor and brain, with the exception of the multiple dose study, which was 4.0 ng/g for brain samples.

### PK characteristics of eribulin in animals

**Fig. 2:**
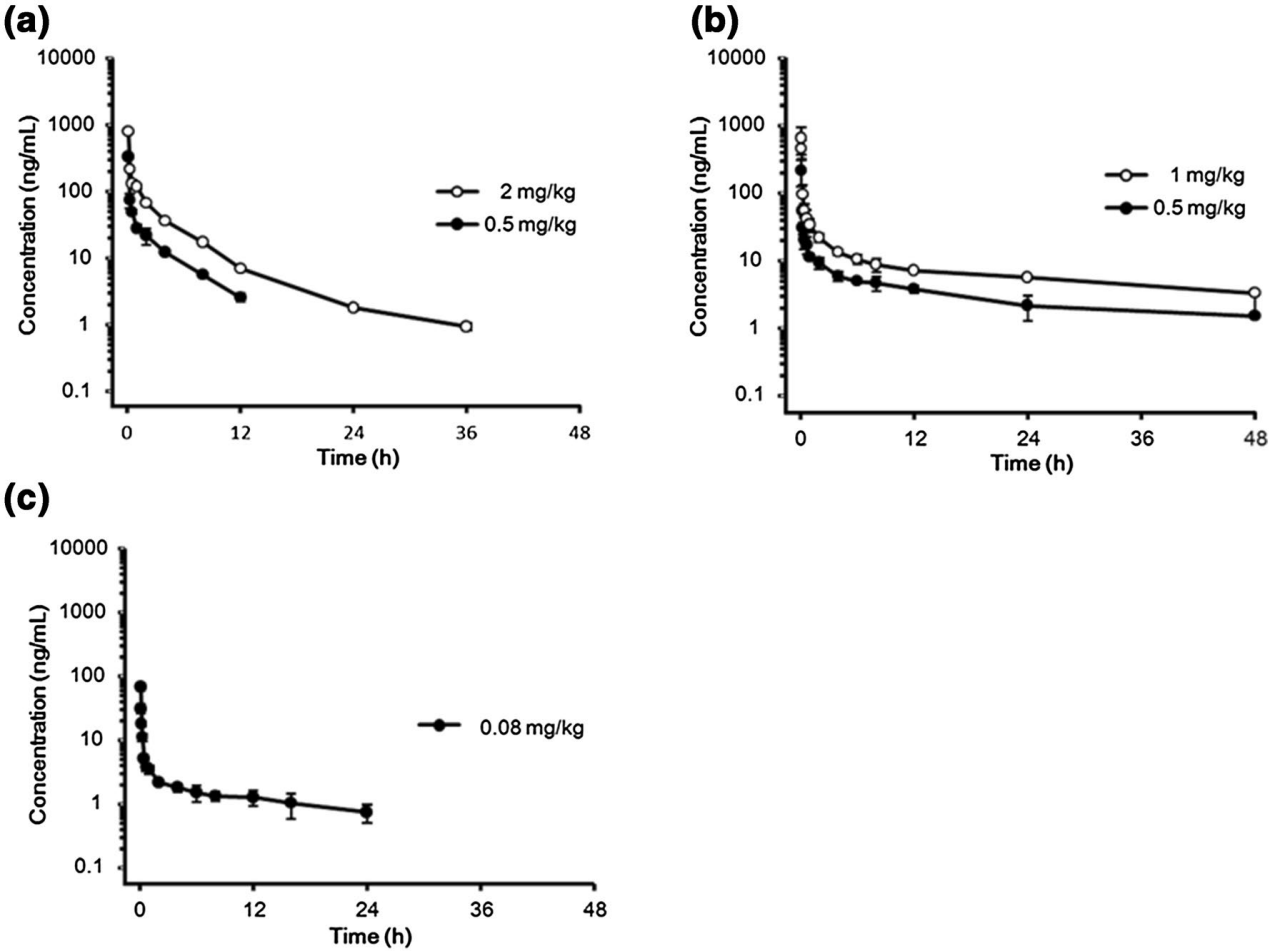
Plasma concentration versus time profiles of eribulin following a single intravenous administration of eribulin. **a** Mouse: data are expressed as mean ± SD (*n* = 3). **b** Rat: data are expressed as mean ± SD (*n* = 3). **c** Dog: data are expressed as mean ± SD (*n* = 4)

Figure 2 shows the mean concentration–time profiles of eribulin in plasma for the three species. PK parameters are listed in Table 1.

**Table 1:** PK parameters of plasma in normal mouse, rat, and dog following a single intravenous administration of eribulin

Parameters	Mouse, *N* = 3/time point		Rat, *N* = 3		Dog, *N* = 4
Dose (mg/kg)	0.5	2	0.5	1	0.08
*t*_1/2_ (h)	3.55	6.88	15.9 ± 7.1	27.9 ± 4.1	21.9 ± 9.5
MRT_inf_ (h)	3.05	4.38	20.2 ± 14.8	27.3 ± 4.1	21.5 ± 11.9
AUC_0–last_ (ng h/mL)	223.2	707.0	212.3 ± 49.3	496.4 ± 80.6	64.5 ± 20.1
AUC_0–inf_ (ng h/mL)	236.4	716.2	259.6 ± 69.6	628.4 ± 83.9	82.2 ± 27.4
CL (L/h/kg)	2.14	2.79	2.04 ± 0.65	1.61 ± 0.20	1.06 ± 0.36
*V*_ss_ (L/kg)	6.44	12.2	36.3 ± 21.0	44.1 ± 10.7	20.4 ± 5.5

Data expressed in mean (for mouse) or mean ± SD (for rat and dog)

The distribution of eribulin was extensive in all animal species tested. In mice, the volume of distribution at the steady state (*V*_ss_) of eribulin in mice was 6.44 or 12.2 L/kg when administered at 0.5 or 2 mg/kg, respectively. The *V*_ss_ at 0.5 mg/kg might be underestimated, because the profile was not characterized properly. In rats, the mean *V*_ss_ averaged 36.3 or 44.1 L/kg when 0.5 or 1 mg/kg of eribulin, respectively, was administered, with no statistical difference between the two doses (*P* = 0.597). In dogs, the mean *V*_ss_ was 20.4 L/kg following administration of 0.08 mg/kg of eribulin, with no major inter-day fluctuation (data not shown).

Total body clearance (CL) of eribulin was moderate in all species tested. In mice, the CL was 2.14 or 2.79 L/h/kg at a 0.5 or 2 mg/kg dose, respectively. In rats, the CL was 2.04 or 1.61 L/h/kg at a 0.5 or 1 mg/kg dose, respectively, with no statistical difference (*P* = 0.335) between the two doses. In dogs, the CL was 1.06 L/h/kg when administered 0.08 mg/kg of eribulin.

Elimination of eribulin was slow in all species studied. In mice, the terminal half-lives (*t*_1/2_) were 3.6 or 6.9 h after 0.5 or 2 mg/kg, respectively. In rats, the *t*_1/2_ were 15.9 or 27.9 h after 0.5 or 1 mg/kg, respectively, and there was no statistical difference between these two doses (*P* = 0.064). Similar values were obtained in dogs. Overall, the results suggest linear pharmacokinetics of eribulin in the dose range tested. Compartmental analysis (data not shown) in general supported the above observation.

### Tumor and brain penetration of eribulin in xenograft model

**Fig. 3:**
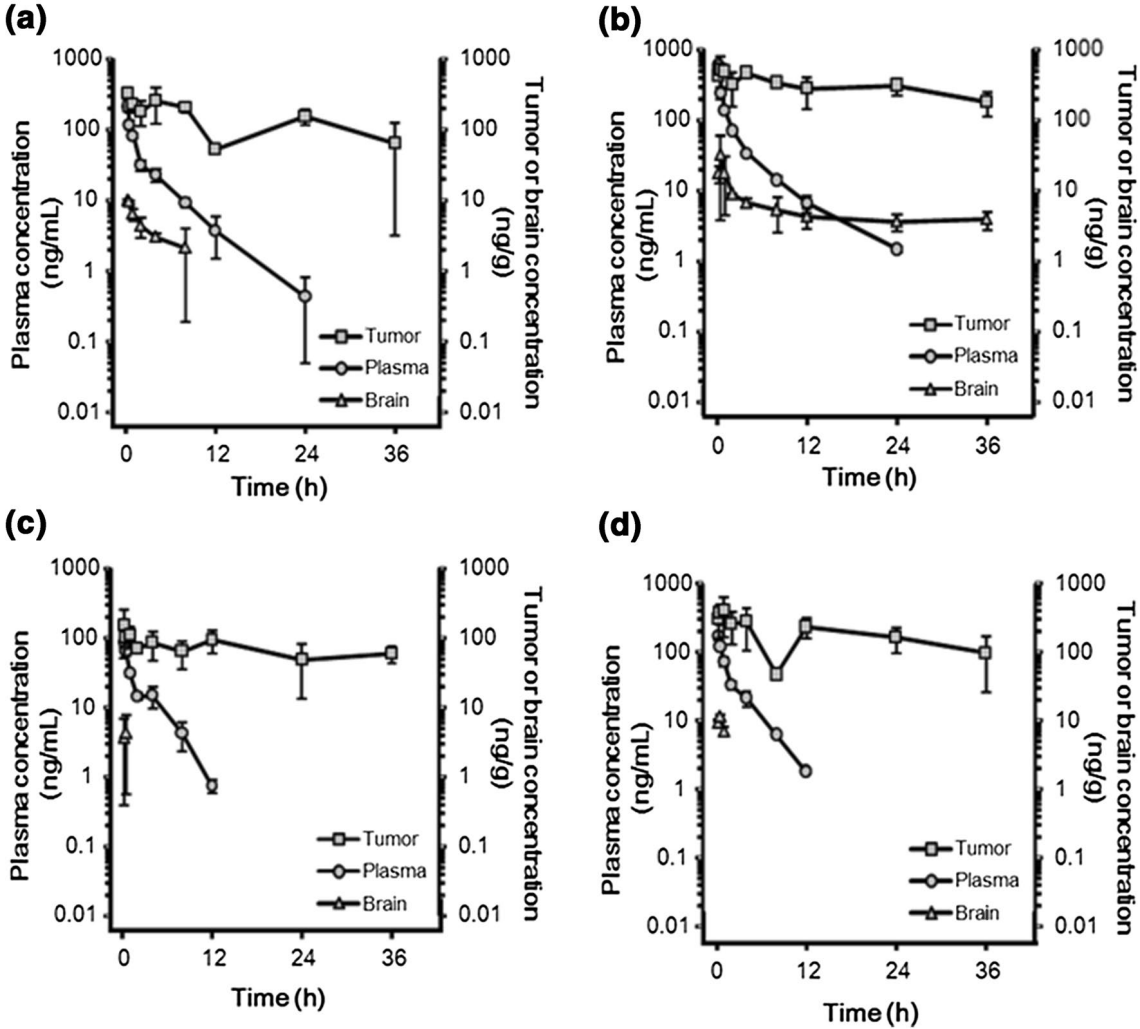
Tumor, brain, and plasma levels of eribulin in LOX human melanoma xenograft model following intravenous administration of eribulin. **a** Single 1.0 mg/kg. **b** Single 2.0 mg/kg. **c** Q2Dx3* 0.5 mg/kg. **d** Q2Dx3* 1.0 mg/kg. *Once every 2 days for three doses. Data are expressed as mean ± SD (*n* = 3)

The data in LOX tumor-bearing mice are summarized in Table 2 and depicted in Fig. 3.

**Table 2:** PK parameters in a LOX human melanoma xenograft model

Dose^a^ regimen	1.0 mg/kg Single dose			2.0 mg/kg Single dose			0.5 mg/kg Q2Dx3^b^			1.0 mg/kg Q2Dx3^b^		
Specimen	Plasma	Brain	Tumor	Plasma	Brain	Tumor	Plasma	Brain	Tumor	Plasma	Brain	Tumor
Parameters												
*C*_max_ (ng/mL or g)	NA	9.75	323.4	NA	31.9	515.3	NA	4.20	153.6	NA	11.5	407.9
*t*_max_^c^ (h)	NA	0.25	0.25	NA	0.5	0.5	NA	0.5	0.25	NA	0.5	1.0
AUC_0-inf_ (ng h/mL or g)	365.2	47.8	6294.3	796.5	361.9	18564.9	161.8	NC	5500.9	308.6	NC	8820.8
AUC_0–inf_/D^d^ (ng h/mL or g/D)	413.2	NA	NA	450.5	NA	NA	366.1	NA	NA	349.1	NA	NA
*t*_1/2_ (h)	3.7	6.0	17.8	5.1	32.4	30.4	2.2	NC	35.9	2.4	NA	19.0
CL (L/h/kg)	2.42	NA	NA	2.22	NA	NA	2.73	NA	NA	2.86	NA	NA
*V*_ss_ (L/kg)	8.03	NA	NA	6.79	NA	NA	7.57	NA	NA	7.41	NA	NA
BPI (AUC_0–inf_^brain^/AUC_0–inf_^plasma^)	0.131			0.454			NC			NC		
TPI (AUC_0–inf_^tumor^/AUC_0–inf_^plasma^)	17.233			23.308			33.999			28.581		

*NA* not applicable, *NC* not calculated due to insufficient data at the terminal phase

^a^Dose of eribulin is expressed in equivalents of the mesylate salt

^b^Once every 2 days for three doses

^c^The time after administration of eribulin when the maximum plasma concentration is reached

^d^D means the free base of eribulin

Plasma PK profiles of eribulin following single and multiple intravenous administrations (Q2Dx3) to LOX tumor-bearing mice were characterized by extensive distribution (*V*_ss_: 6.79–8.03 L/kg), moderate clearance (CL: 2.22–2.86 L/h/kg), and moderate elimination (*t*_1/2_: 2.2–5.1 h).

Penetration of eribulin into the tumors was fast (*t*_max_ = 0.25–1.0 h). The calculated TPI showed that exposures to eribulin were approximately 20–30 times higher in tumor than in plasma after both single and multiple dose administration. The TPI following multiple dosing was slightly higher than that observed after single dosing, but it is not possible to conclude that there was accumulation of eribulin in the tumor after multiple dosing because of the variability associated with estimating *t*_1/2_ and AUC from time 0 to infinity (AUC_0–inf_).

The BPI of eribulin in mice was poor, with exposures ranging from approximately 13–45% of those observed in plasma after a single dose administration.

## Discussion

PK profiles of eribulin in mice, rats, and dogs were characterized by high volume of distribution, moderate clearance, and slow elimination. The clearance was similar in the three species. In xenograft female mice, the parameters were similar to those of male BALB/c mice. However, the volume of distribution showed species differences; it was smaller in mice compared with rats and dogs. The reason behind the species differences is uncertain. The disposition profile of eribulin was biphasic, with a rapid distribution phase (within minutes) followed by a long elimination phase. The eribulin half-lives were shorter in mice compared with rats and dogs. The high volume of distribution suggests that eribulin is extensively distributed extravascularly. This is expected due to low protein binding of eribulin and its good membrane permeability. Overall, the PK profiles are similar between animals and humans [11, 12, 19].

In a preclinical study, eribulin showed wide in vivo therapeutic windows [1]. We investigated eribulin exposure in tumor and brain using a xenograft model. Efflux transporters, such as P-gp, are expressed in the blood–brain barrier and in cancer cells, which is one of the expected reasons for resistance against anti-cancer chemotherapy [20]. Eribulin is likely to be a P-gp substrate as it shows greater brain penetration in ABCB member1a-deficient mice than that in wild-type mice [15]. In the present study, the BPI in xenograft mice showed low distribution in brain compared with that in plasma, indicating that eribulin is a P-gp substrate and would have a little effect in the central nervous system. On the other hand, the TPI of eribulin indicated that tumor exposure was approximately 20–30 times higher than that in plasma after both single and multiple dose regimens.

Tumor penetration of other tubulin-binding drugs, such as paclitaxel, in xenograft mice has been well studied. Hamaguchi reported that the TPIs of paclitaxel in colon tumor-bearing mice were 1.07 and 1.49 [21] and those of eribulin in our study were 17.2–33.9. The half-lives of paclitaxel and eribulin in tumor were 7.02–8.06 and 17.8–35.9 h, respectively. These results suggest that eribulin has higher tumor retention than paclitaxel; however, a direct comparison cannot be made as these are different studies evaluating distinct tumor types.

The mechanism of retention in tumor is a balance between distribution and elimination, and depends on both tumor- and drug-specific parameters [22, 23]. Among drug-specific characteristics, a possible reason to explain the high tumor retention of eribulin is related to its mechanism of action. Eribulin showed irreversible mitotic block leading to apoptosis and complete loss of long-term viability at 5 days postwashout [2]. Thus, once eribulin reaches the target tumor cells and binds to high-affinity sites, it will be retained inside the tumor and have persistent activity. Towle et al. also reported that paclitaxel is classified as a drug with reversible activity, suggesting that paclitaxel and eribulin have distinct mechanisms [2].

Additional mechanisms may contribute to a higher distribution of eribulin into tumor tissues. Eribulin was reported to have an effect on vascular normalization and reoxygenation. Eribulin-induced remodeling of vasculature leads to uniform tumor perfusion across all tumor regions in human breast cancer [6] and soft tissue sarcoma [24]. Tumor microenvironment is abnormal compared with that of nonmalignant tissues, and eribulin-induced increased tumor perfusion increased the ability of subsequently administered drugs to reach tumor areas that had previously been poorly perfused. Overall, the high TPI of eribulin, in combination with its diverse mechanism of action, would account for its balanced profile of efficacy and safety.

In the clinical study, eribulin also showed longer activity in tumor [19]. Significant morphologic changes (bundle formation), induced by eribulin, were observed in the microtubules of peripheral blood mononuclear cells and tumor cells in vivo for at least 24 h, and the plasma concentrations of eribulin are maintained well above the levels required for the activity in vitro (sub-nM) for >72 h [19]. Regarding retention of higher concentration, the limited metabolism of eribulin would contribute to slow elimination in humans. Furthermore, the vascular remodeling effect of eribulin observed in preclinical studies was also confirmed in clinical studies. Optical imaging technology revealed that oxygen saturation was increased in tumors after eribulin infusion, suggesting that eribulin induces tumor reoxygenation and perfusion [5]. Thus, the recovery of tumor perfusion might also contribute to longer retention of eribulin in human.

In conclusion, eribulin was distributed rapidly and eliminated slowly in mice, rats, and dogs. In female xenograft mice, eribulin PK parameters were similar to those of male BALB/c mice. Eribulin exposure was approximately 20–30 times higher in tumor than in plasma after both single and multiple intravenous dose administrations. These results might be caused by eribulin’s mechanism of action including increased perfusion in tumor by due to the remodeling effect. The results of the present study may help to explain the well-balanced efficacy and safety of eribulin in clinical studies.

## Abbreviations

ABCB: ATP-binding cassette subfamily B
ABCC: ATP-binding cassette subfamily C
AUC: Area under the plasma concentration versus time curve
CL: Total body clearance
*C*_max_: Maximum plasma concentration
IS: Internal standard
LC–MS/MS: Liquid chromatography–tandem mass spectrometry
MRT_inf_: Mean residence time extrapolated to infinity
*m/z*: Mass-to-charge ratio
P-gp: P-glycoprotein
PK: Pharmacokinetic
Q2Dx3: Once every 2 days for three doses
*t*_1/2_: Terminal half-life
*V*_ss_: Volume of distribution at the steady state
